# A comprehensive dataset of *Avena sativa* L. landraces phenotypes and genotype

**DOI:** 10.1016/j.dib.2022.107962

**Published:** 2022-02-16

**Authors:** Dorota Dziubińska, Paulina Bolc, Grzegorz Kloc, Wiesław Podyma, Maja Boczkowska

**Affiliations:** Plant Breeding and Acclimatization Institute (IHAR) - National Research Institute, Radzików, Błonie 05-870, Poland

**Keywords:** Common oat, Evaluation data, Agronomic traits, Molecular markers

## Abstract

The world germplasm collection of the genus *Avena* contains about 130,000 accessions, of which more than half are cultivated forms. Collection and sharing of passport data and phenotypic traits is carried out by most gene banks. However, genetic data for the stored accessions are rarely collected and provided. We present a composite data set consisting of passport data, description of agro-morphological traits, and genotypic data for 54 accessions preserved in the Polish gene bank. All accessions are unique landraces collected during expeditions in Poland. Phenotypic data were obtained from a minimum of 3 years of field trials. Genotypic data resulted from Inter Simple Sequence Repeat (ISSR) markers analysis. The data set was supplemented with meteorological data from a meteorological station located no more than 2 km in a straight line from the experimental plots. This data set can be used in meta-analyzes of germplasm collection diversity data. These data can be valuable to researchers and breeders looking for new sources of variation. The data were used in the research article “Promoting the Use of Common Oat Genetic Resources through Diversity Analysis and Core Collection Construction” [1].

## Specifications Table

**Table:** 

Subject	Agricultural science, Agronomy and Crop science
Specific subject area	Common oat binary genetic data, agro-morphologic data based on 3-years trials, accession passport data, meteorological data
Type of data	Table
How the data were acquired	Genotyping data were collected by PCR (Applied Biosystems Veriti 96-Well Thermal Cycler) with DNA isolated from oat accessions, and then analysis in ABI 3130XL Genetic Analyzer (Applied Biosystems). Agro-morphological data were obtained by at least 3-year field observation. Meteorological data were collected by a meteorological station in the Plant Breeding and Acclimatization Institute (IHAR) - National Research Institute, Radzików 05–870 Błonie, Poland.
Data format	Raw
Description of data collection	54 common oat landraces preserved in the National center for Plant Genetic Resources. Phenotypic traits were evaluated in a minimum of three-year field experiments. DNA was extracted from young healthy leaves separately from 24 individuals representing each of 54 accessions. Genetic analysis was performed using eight Inter Simple Sequence Repeat (ISSR) primers using a capillary sequencer.
Data source location	Plant Breeding and Acclimatization Institute (IHAR) - National Research Institute, Radzików 05–870 Błonie, Poland
Data accessibility	Repository name: Open Science Framework Data identification number (permanent identifier, i.e. DOI number): 10.17605/OSF.IO/DJ8GE Direct link to the dataset: https://osf.io/dj8ge/
Related research article	M. Boczkowska, B. Łapiński, I. Kordulasińska, D.F. Dostatny, J.H. Czembor, Promoting the use of common oat genetic resources through diversity analysis and core collection construction. Plos one, 11 (2016)., e0167855. https://journals.plos.org/plosone/article?id=10.1371/journal.pone.0167855

## Value of the Data

• We provide data on the genotypic and phenotypic diversity of oat landraces collection preserved in the National center for Plant Genetic Resources. It should be noted that these are data for landraces, the seeds of which can be obtained free of charge from the Polish gene bank for further research and breeding according to the Standard Material Transfer Agreement. Comprehensive data provide enhanced usage of genetic resources for breeding and scientific purposes.
• The data can be used by researchers and breeders interested in new sources of genetic variation.
• The data can be used to select accessions with specific phenotypic characteristics and a specific genetic background. It can also be used for meta-analysis of genetic variation.

## 1 Data Description

All data refer to a set of 54 A*. sativa* accessions from the collection preserved at the National Center for Plant Gene Resources, i.e., the Polish gene bank. Seeds of these accessions are available for science and breeding with the approval of SMTA.

The tab-delimited text file passport_data.txt contains information about the accession set. The following information is included in each row: Institute code (Plant Breeding and Acclimatization Institute (IHAR) - National Research Institute code POL003), Accession number, Genus, Species, Species author, Sub taxa, Sub taxa author, Accession name, Acquisition Date, Country of origin, Collection site, Collection site latitude, Collection site longitude, Collection site elevation (value in mamsl), Sample Status, Duplicate storage site (code of, Svalbard Global Seed Vault, NOR051) Storage type, Accession URL (direct link to NCPGR database site), Crop Groups, Rights of third parties (0 mean – free from third parties rights), MLS status, Curator name. A blank space means no data. The subsequent rows correspond to the accessions.

The tab-delimited file phenotypic_data.txt contains data from field experiments conducted between 1981 and 2021. The following information is included in each row: National Inventory code, Institute code, Accession number, Evaluation year, Evaluation country, Sowing date, Heading date, Panicle length [cm], Plant hight [cm], Lodging resistance, Powdery mildew resistance, Crown rust resistance, Stem rust resistance, Septoria resistance, BYDV, Yield [g], 1000 grain weight [g]. A blank space means no data. Subsequent rows represent data from the evaluation of one accession in one year.

The tab-delimited file genotypic data.txt contains data from ISSR analysis. The first row contains headings i.e., Accession (accession number from the gene bank), Plant (number of seedlings tested), further there are headings describing the obtained fragment recorded in the format primer name_fragment length in base pairs. Subsequent lines contain data on the presence of the fragment coded as 0 - no fragment, 1 - presence of the fragment.

The excel sheet weather_data.xls contains meteorological data collected by meteorological station located in the Plant Breeding and Acclimatization Institute (IHAR) - National Research Institute. The following sheets contain data (Mean air temperature andTotal precipitation) for each day from 2005 to 2020. Additionally, there is a min-max sheet containing minimum and maximum monthly temperatures for the period 2006–2020. The last sheet contains extended weather data for the period 2009–2020. It contains information about Air temperature, Total precipitation, Solar Insolation, Atmospheric pressure, Dew point temperature, Frost point temperature, Solar radiation, Net radiation, Relative humidity, and Soil temperature.

## 2 Experimental Design, Materials and Methods

### 2.1 Plant material

The plant material consisted of 54 common oat accessions preserved in the National center for Plant Genetic Resources i.e., Polish gene bank. The accessions have landrace/traditional cultivar status and were collected during expeditions in Poland. Accessions were included in the gene bank collection between 1973 and 2001.

### 2.2 Evaluation of agro-morphologic traits

Phenotypic traits were evaluated in a minimum of three-year field experiments on 2.5 m^2^ plots to which 600 seeds were machine sown. Neither fertilization nor chemical protection against pathogens was applied in the fields. The evaluation was conducted on experimental fields of the Plant Breeding and Acclimatization Institute (IHAR) - National Research Institute in Radzików, 05–870 Błonie, Poland (52.2165, 20.6453). Depending on the weather conditions, sowing was carried out from mid-March to mid-April. Plant height was determined at the seed ripening stage. Measurements of height from the ground to the top of panicle were taken for 10 randomly selected plants per plot. The arithmetic mean was taken from the obtained measurements. At the same time the length of panicle measured from the base to the tip was determined for 10 randomly chosen plants. The arithmetic mean was calculated from the obtained measurements. During the vegetation season natural infection of diseases such as crown rust, stem rust, powdery mildew, septoria, and barley yellow dwarf virus (BYDV) were observed. The observations were carried out on two separate dates, i.e., at the shooting stage and at the heading time. The resistance of cultivars to disease infection was evaluated on a 9-grade scale, where 1 means the maximum intensity of the trait (complete disease infection), and 9 means no occurrence of the disease. At grain ripening stage, two observations of lodging were made at an interval of three weeks. The resistance to lodging was determined on a 9-grade scale, where 1 means maximal intensity of the trait, and 9 lack of its occurrence. The grain was mechanically harvested. Grain yield was determined after thorough drying and removing plant residues. The yield was measured in kilograms per plot. The weight of one thousand grains was estimated by weighing three repetitions of 100 grains each, calculating the average and multiplying it by 10.

### 2.3 DNA extraction

In the greenhouse, 50 seeds from each *A. sativa* accession were sown into 60 mm diameter pots filled with peat substrate. Tissue from young healthy leaves was obtained at the second leaf stage. Each accession was represented by 24 individuals. Tissue from each individual was collected into separate 2.0 ml Eppendorf type tubes, lyophilized, and grinded in a bead mill (Mixer Mill MM 200, Retsch, Haan, Germany). DNA was isolated using Genomic Mini AXE Plant kit (A&A Biotechnology) according to the manufacturer's protocol. DNA concentration and purity were determined spectrophotometrically (NanoDrop ND-1000, NanoDrop Technologies, Willmington, DA, USA). Integrity was assessed visually by electrophoresis in a 1.5% agarose gel in the presence of ethidium bromide. DNA isolates were then diluted to a working concentration of 25 ng/µl.

### 2.4 Genetic analysis

Genetic analysis was performed using Inter Simple Sequence Repeat (ISSR) markers. The 10 ul PCR reaction mixture consisted of 25 ng of genomic DNA, 1 u SuperHotStart Taq polymerase (Bioron), 1x Taq buffer, 1.9 mM MgCl2, 0.2 mM dNTP's mix and 1.5 µM primer and was performed in a thermocycler Verity 96 Thermal Cycler (Applied Biosystems) under the following temperature profile: 5′ - 94 °C followed by 45 cycles: 30″ - 94 °C, 45″ - 58 or 54 °C, 2′ - 72 °C and the final extension for 10′ - 72 °C. A set of 8 ISSR primers (Table 1) anchored at the 5′ or 3′ end that were selected in a previous study were used [2]. The high efficiency of this set of markers was also confirmed in other studies [3], [4], [5], [6], [7], [8], [9], [10]. Primers were fluorescently labelled at the 5′ end. The PCR reaction products were separated and visualized using a capillary sequencer ABI 3130xl Genetic Analyzer (Applied Biosystem), using a 16-capillary array filled with POP-7 polymer (Applied Biosystem). The lengths of the fragments were assessed in relation to the size standard GeneScan1200 LIZ Size Standard (Applied Biosystems).

**Table 1:** ISSR primers information.

Primer	Sequence	Dye	Optimal Tm °C
UBC 807	(AG)8T	6-FAM	58
UBC 825	(AC)8T	6-FAM	58
UBC 834	(AG)8YT	VIC	54
UBC 841	(GA)8YC	VIC	54
UBC 856	(AC)8YA	NED	54
UBC 857	(AC)8YG	NED	54
UBC 884	HBH(AG)7	PET	54
UBC 885	BHB(GA)7	PET	54

### 2.5 Assembling meteorological data

Meteorological data from 2005 to 2020 concerning at least temperature and total precipitation data were collected daily by the meteorological station located on the area of the Plant Breeding and Acclimatization Institute (IHAR) - National Research Institute up to two km in a straight line from experimental plots. For the years 2006–2020, minimum and maximum monthly temperature data were also collected along with the date of their occurrence. Extended meteorological data were also collected for 2009–2020.

## Ethics Statements

Not applicable.

## CRediT authorship contribution statement

**Dorota Dziubińska:** Investigation, Data curation, Writing – original draft. **Paulina Bolc:** Writing – original draft. **Grzegorz Kloc:** Data curation. **Wiesław Podyma:** Supervision, Resources, Funding acquisition. **Maja Boczkowska:** Conceptualization, Investigation, Methodology, Writing – review & editing.

## Declaration of Competing Interest

The authors declare that they have no known competing financial interests or personal relationships that could have appeared to influence the work reported in this paper.
